# How Neutrophil Extracellular Traps Become Visible

**DOI:** 10.1155/2016/4604713

**Published:** 2016-05-16

**Authors:** Nicole de Buhr, Maren von Köckritz-Blickwede

**Affiliations:** ^1^Department of Physiological Chemistry and Department of Infectious Diseases, University of Veterinary Medicine Hannover, Bünteweg 17, 30559 Hannover, Germany; ^2^Research Center for Emerging Infections and Zoonoses (RIZ), University of Veterinary Medicine Hannover, Bünteweg 17, 30559 Hannover, Germany

## Abstract

Neutrophil extracellular traps (NETs) have been identified as a fundamental innate immune defense mechanism against different pathogens. NETs are characterized as released nuclear DNA associated with histones and granule proteins, which form an extracellular web-like structure that is able to entrap and occasionally kill certain microbes. Furthermore, NETs have been shown to contribute to several noninfectious disease conditions when released by activated neutrophils during inflammation. The identification of NETs has mainly been succeeded by various microscopy techniques, for example, immunofluorescence microscopy, transmission electron microscopy (TEM), and scanning electron microscopy (SEM). Since the last years the development and improvement of new immunofluorescence-based techniques enabled optimized visualization and quantification of NETs. On the one hand* in vitro* live-cell imaging led to profound new ideas about the mechanisms involved in the formation and functionality of NETs. On the other hand different intravital,* in vivo*, and* in situ* microscopy techniques led to deeper insights into the role of NET formation during health and disease. This paper presents an overview of the main used microscopy techniques to visualize NETs and describes their advantages as well as disadvantages.

## 1. Introduction

Neutrophils are phagocytic cells, which are an essential part of the innate immune defence against invading pathogens. They have the ability to rapidly infiltrate infected tissue after breaching stabilizing cell barriers [1, 2]. In 2004, the formation of neutrophil extracellular traps (NETs) was identified as a novel extracellular strategy of neutrophils to combat different invading pathogens [3]. NET structures consist of decondensed chromatin released by activated neutrophils in response to infection and inflammation (reviewed by [4]). Factors known to induce NETs are cytokines like IL-8 or TNF*α* or various pathogens [3]. Histones, antimicrobial peptides (AMPs), and granule proteins are bound to the decondensed chromatin and form web-like structures which mediate entrapment of invading microbes. Extracellular NET-mediated entrapment has been shown for parasites [5, 6], viruses [7–9], fungi [10–12], and most often for bacteria (reviewed by [11]). This occasionally can contribute to growth inhibition or killing of the pathogen by the host immune system. Furthermore, NETs have been shown to contribute to noninfectious diseases, for example, chronic diseases, autoimmune diseases, thrombosis, or cancer [3, 13–15]. The key findings that have proven an essential role of NETs in health and disease were mainly accomplished by microscopy. Since the first identification of NETs in 2004, different* in vitro* and* in vivo* microscopy methods and staining protocols were published. In this review an overview of the main methods and developments used to visualize NETs is summarized with their advantages and pitfalls (Table 1).

## 2. Neutrophil Extracellular Traps (NETs)

### 2.1. Composition of NETs and Their General Function

The hallmark of the formation of NETs is the extracellular release of DNA associated with antimicrobial compounds. These antimicrobial components are myeloperoxidase (MPO), neutrophil elastase (NE), cathelicidins like LL-37, histones, proteinase 3, cathepsin, lactoferrin, or gelatinase [3, 16, 17]. With those compounds NETs are able to bind, disarm, and occasionally inhibit the growth or kill various pathogens. Importantly, a recent study on gout, an acute inflammatory reaction in response to monosodium urate crystals, described an additional function of NETs in the innate immune system [14]: aggregated NETs (aggNETs) are able to degrade cytokines and chemokines via serine proteases, which are formed during inflammatory processes when a high neutrophil density is present. During acute gout, aggNETs constitute an anti-inflammatory mechanism and reduce the recruitment and activation of neutrophils. NETosis-deficient mice develop exacerbated and chronic disease that can be reduced by adoptive transfer of aggNETs [14].

However, besides these two main protective effects of NETs, entrapment of pathogens and degradation of cytokines or chemokines, increasing evidence is accumulating which describes detrimental consequences for the host when NETs are inefficiently eliminated, for example, in various autoimmune diseases, such as systemic lupus erythematosus (SLE) and systemic vasculitis (SVV), autoimmune lupus nephritis, or rheumatoid arthritis [18–22]. Therefore, a constant fine balance between NET formation and its subsequent degradation is needed to accomplish protective effects during infection and inflammation. The key regulators of NET degradation are host DNases. An impairment of NET degradation by missing or nonfunctional nucleases is associated with autoimmune diseases, for example, lupus nephritis [20]. Accordingly, it was demonstrated that nuclease-deficient individuals are more prone to SLE.

**Figure 1:**
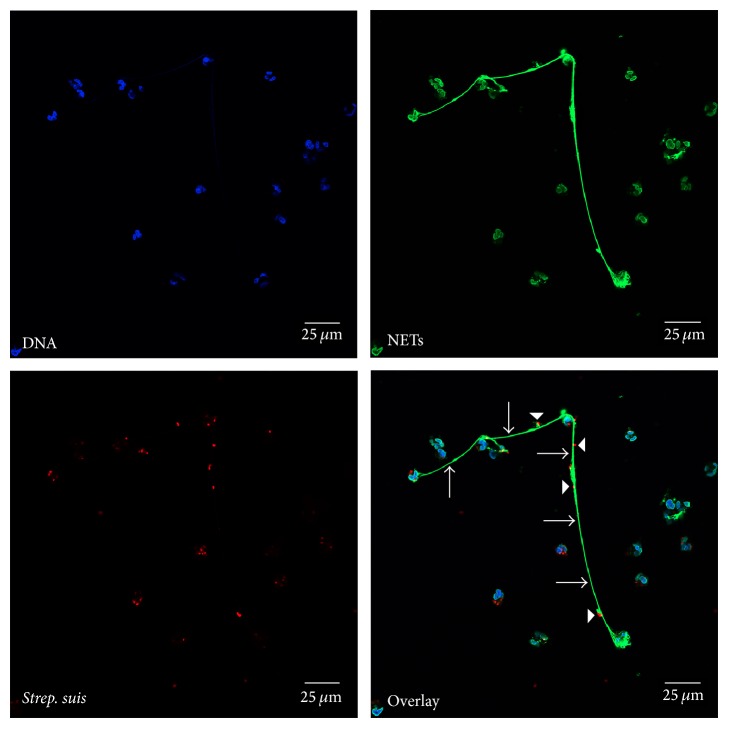
NETs entrapping* Streptococcus (Strep.) suis *Δ*endAsuis *Δ*ssnA*. Immunofluorescence microscopy analysis of NETs released by human neutrophils 4 h after infection with the DNase deletion mutant* Strep. suis *Δ*endAsuis* Δ*ssnA in vitro* [25]. Neutrophils and bacteria were centrifuged on poly-L-lysine-coated coverslips and the nuclei were stained with Hoechst (blue). Furthermore, the samples were incubated with a mouse monoclonal antibody against DNA/histone 1 (green, arrows) visualizing long extracellular fibres of released NETs and rabbit anti-*Strep. suis* antibody to label entrapped* Strep. suis* (red, arrowheads). The secondary staining was performed with goat anti-mouse Alexa 488-conjugated antibody and goat anti-rabbit Alexa 633-conjugated antibody. The coverslip was embedded in Prolong® Gold antifade. Samples were recorded using a Leica TCS SP5 confocal inverted-base fluorescence microscope with a HCX PL APO 40x 0.75–1.25 oil immersion objective. Settings were adjusted with control preparations using a respective isotype control antibody.

After DNase digestion the antimicrobial activity of NETs is lost [3]. Therefore, it was assumed that DNase production is a benefit for pathogens and used as microbial host evasion factor to avoid entrapment by NETs as first of all described for group A streptococci (GAS) [23]. Nowadays, the production of DNases as NET evasion molecule has been described for numerous pathogens, thereby enabling escape of microbes from NETs. Most DNases identified as a NET evasion factor are produced by Gram-positive cocci and, interestingly, some of them produce more than one DNase like in case of* Streptococcus suis* the nucleases SsnA and EndAsuis (Figure 1) or in case of group A* Streptococcus* SdaD2, Spd, and Spd3 [24–26], for example. There is increasing evidence that also Gram-negative bacteria like* Neisseria gonorrhoeae*,* Vibrio cholera*,* Yersinia enterocolitica*, or* Aeromonas hydrophila* are also able to escape from NETs by production of DNases, although the exact role of NETs during pathogenesis upon infection with those pathogens is still not entirely clear [27–30].

### 2.2. Mechanisms of NET Formation: Vital versus Suicidal NETosis

Most publications describe NET release as a form of pathogen-induced active cell death, which gives neutrophils the possibility to fight against microbes beyond their life span. Classically, NETosis is the term used for the release of NETs by dead cells [2]. NETosis is described as a novel cell death of neutrophils besides apoptosis and necrosis which is characterized by the disruption of the nuclear membrane allowing mixing of nuclear and granule components that are released as web-like fibers to the extracellular space [1, 31–33]. For the induction of NETosis, neutrophils can be activated by different physiological stimuli, for example, bacteria. The most frequently used chemical stimulator for human blood-derived neutrophils to release NETs is phorbol-12-myristate-13-acetate (PMA). After this the neutrophils undergo typical features of NETosis and release NETs in a time span of two to three hours as monitored by Fuchs et al., using live video microscopy [34]. It was summarized that this mechanism of NET release during cell lysis is initiated after a direct oxidative burst-dependent neutrophil activation by several pathogens [16].

**Figure 2:**
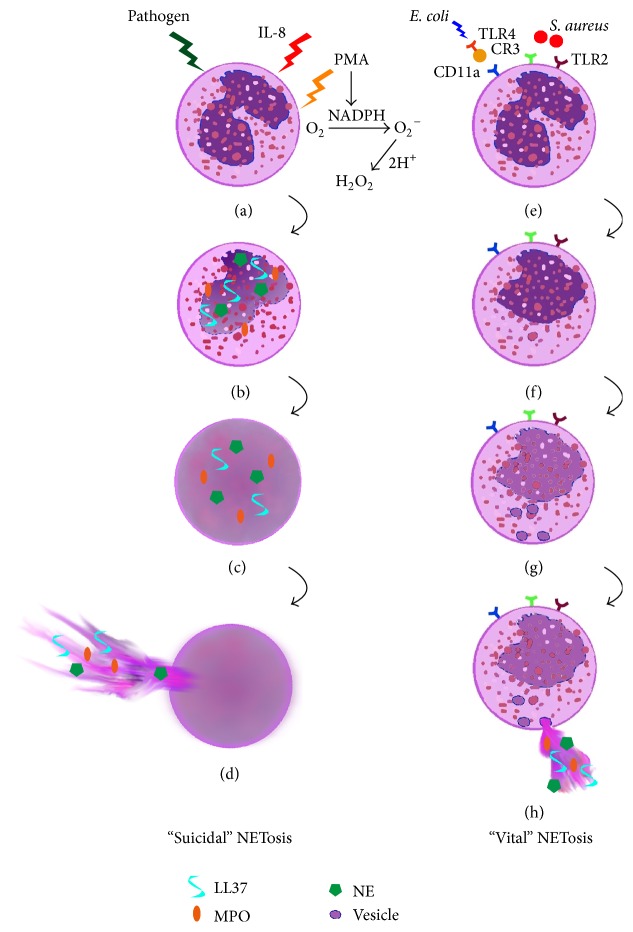
Mechanisms of NET formation: vital versus suicidal NETosis. (a)–(d) The “suicidal” NETosis starts after a stimulation by, for example, PMA, IL-8, or various microbial pathogens in a NADPH-oxidase-dependent matter and leads to NET release within 1 to 4 h. Translocation of MPO, elastase, and LL-37 to the nucleus leads to the nuclear decondensation and disruption of the nuclear membrane [78, 79]. Subsequently, the content of the nucleus mixes with the granular as well as cytosolic proteins. Finally, the outer membrane ruptures and NETs are released by the activated neutrophils into the extracellular space. (e)-(f) The “vital” NETosis has been described to be a rapid release of NETs (5–60 min). It can be induced by a TLR-4-mediated platelet activation and its interaction with CD11a on neutrophils [38]. Furthermore, an activation via complement receptor 3 (CR3) and TLR-2 has also been shown in the presence of Gram-positive bacteria, for example,* Staphylococcus (S.) aureus* [35]. The nucleus becomes rounded and decondensed (f). Vesicles with nuclear DNA are formed (g) and NETs are released via nuclear budding (h). The outer membrane remains intact upon NET release and the anuclear neutrophil retains the ability to multitask [35] (adapted [36]).

In 2012 a groundbreaking study on the* in vivo* relevance of NETs was published in Nature Medicine, where an intravital live imaging technique was used to visualize the process of NET formation* in vivo* [35]. The authors demonstrated that neutrophils released NETs during the course of an acute infection with* Staphylococcus aureus*, but those neutrophils were viable and still able to phagocytose and crawl. Based on this finding, the authors discussed that a population of neutrophils is still able to retain the ability to multitask when releasing NETs in response to an infection [35]. Thus, a suicidal and a vital NETosis can be differentiated (Figure 2) as also reviewed by Yipp and Kubes 2013 [36]. The vital NETosis is, in contrast to the suicidal NETosis, characterized by a rapid vesicular release of nuclear DNA within minutes upon stimulation of neutrophils [35, 37]. In good accordance with this, an earlier study already demonstrated that neutrophils which release NETs upon contact with TLR-4-activated platelets still remain intact, since the access of SYTOX dye to the NET-releasing neutrophils was restricted [38]. SYTOX dyes can penetrate only damaged cell membranes and are thereby an indicator for cell death. Furthermore, this fast vesicular NET release (5–60 min) was also identified in a TLR-2 and complement receptor 3 (CR3) dependent way [35], whereas in the suicidal NETosis the plasma membrane is described to be disrupted during the NET release; this is not the case for this newly described vital NETosis. In case of vital NETosis, the multilobular nucleus first becomes quickly rounded and decondensed. Afterwards the nuclear envelope degrades and chromatin is visible in the cell center. DNA containing vesicles concentrate near the plasma membrane and finally NETs are released without disruption of the plasma membrane. A further difference compared to the suicidal NET release is that the vital NET release seems to be oxidative burst independent [35–37]. Finally, as a poorly understood third mechanism of NET formation, the release of mitochondrial DNA by viable neutrophils was described [39].

In summary, there is increasing evidence that NET formation may occur by three different mechanisms [3, 34, 37, 39]: (1) classical NET release through cell lysis (NETosis); (2) NET release by viable cells mediated by a vesicular release of nuclear DNA; and (3) NET release by viable cells formed of mitochondrial DNA. Importantly, the vital NETosis via vesicular release of nuclear DNA seems to be faster and oxidative burst independent, but the detailed cellular mechanisms that lead to NET formation by viable cells or by release of mitochondrial DNA are still not entirely clear.

## 3. *In Vitro* NET Visualization Techniques

### 3.1. General Steps before Staining of NETs

For NETs visualization the nonadherent neutrophils are normally seeded on poly-L-lysine-coated glass coverslips. Therefore, ready-to-use poly-L-lysine-coated glass coverslips can be used or the coating can be performed using commercially available poly-L-lysine solutions [40]. Protocols regarding an optimal cell density for good visualization results and NET quantification are available [40, 41]. For NET quantification, two main parameters are used in the literature (also see Table 1): (1) the NET induction measured by quantifying percentage of NET release or amount of NETs per field of view and (2) the NET degradation measured by quantifying area of NETs. The NET induction measuring percentage of NET formation compared to respective negative control is mainly used to identify NET-inducing agents or to characterize mechanisms involved in NET formation over time. An overview of NET-activating agents was already given in a review published 2012 by Goldmann and Medina [42]. Besides, in some animal species other NET inducers are known to be very effective, for example, zymosan in neutrophils derived from cattle [6]. The NET degradation assay which measures area of NETs is mainly used to characterize NET-eliminating nucleases, for example, for the identification of new microbial nucleases as NET evasion factors. One important key step in all experiment is the selection of respective negative and positive controls, especially since the NET formation by human blood-derived neutrophils is highly donor dependent [34]. Thus, in case of studying NET induction a negative control without any stimulation is always needed and shows the background or spontaneous NET formation by the neutrophil population of the respective donor.

At the end of the experiment, the samples are normally fixed with 4% paraformaldehyde (PFA) and can be stored at 4°C for further analysis, for example, fluorescence microscopy (see below). Finally, it has to be mentioned that for all experiments a contamination of washing reagents or tissue culture media with nucleases, which, for example, may be present in bovine serum albumin or fetal calf serum, needs to be avoided, since this may cause artificial results as previously reported [43].

### 3.2. Fluorescence Microscopy to Visualize NETs

A summary of the main NET visualization techniques used for quantification of NET degradation or induction is given in Table 1. Since the major backbone of NETs is the DNA, different DNA-intercalating dyes, for example, Dapi, propidium iodide, SYTOX Orange, or SYTOX Green, are widely used to visualize NETs. However, since histones and several granule components, for example, myeloperoxidase or elastase, have also been shown to be present at high amounts within NETs, additional immunostaining of those enzymes with respective antibodies can help to visualize NETs. Importantly, we have recently shown that cationic antimicrobial peptides, for example, the cathelicidin LL-37, which are associated with NETs, block the binding of DNA-intercalating dyes to the NETs and thereby hamper its visualization [44]. Therefore, the antibody-based techniques should always be the first choice to visualize and quantify NETs. However, fluorescence microscopy has two main limitations: the results may be biased by the observer and it does not allow rapid screening of larger number of cells or samples. Several authors described automated systems to facilitate quantification of the results obtained by fluorescence microscopy (as summarized in Table 1), which may help to optimize a fluorescence microscopy-based quantification of NET formation or NET degradation.

### 3.3. Fluorescence Spectroscopy to Quantify NETs

In addition to the visual assessment of NET formation by fluorescence microscopy, a spectrofluorometric method is often used to quantify NETs in a more high-throughput application. This method is also based on the fact that DNA is the major backbone of NETs. Some authors simply use SYTOX dyes to stain all dead cells and released DNA of a neutrophil population as an indicator for NETosis (Table 1). In an optimized assay, micrococcal nuclease is used to disrupt the NETs and to release the DNA of NETs into the supernatant. The amount of extracellular DNA can then be quantified in the supernatant of cells by using PicoGreen (Invitrogen). PicoGreen is an ultrasensitive fluorescent nucleic stain for quantifying double-stranded DNA (dsDNA) in solution. The percentage of released NET-DNA can be calculated using total cell DNA as 100%. Importantly, we have previously shown that the PicoGreen assay is not sensitive enough to detect a relatively small amount of neutrophils (< than 20% within 1 h after stimulation) that are releasing NETs. In this case, the antibody-based microscopic evaluation of NET release is more sensitive [40]. However, as mentioned above, the PicoGreen assay is a useful tool to investigate the formation of NETs in a more high-throughput format. Nevertheless, a microscopic confirmation of the results is necessary to exclude necrotic release of cellular DNA and to thus confirm specificity of the assay [40].

### 3.4. Flow Cytometry to Visualize NETs

Recently, two flow cytometry-based techniques to determine NETs have been characterized: Gavillet et al. described a flow cytometry-based model using antibodies against two key components of NETs, specifically citrullinated histones and myeloperoxidase in combination with DNA dyes [45]. The method was validated by comparison with the well-established antibody-based microscopy assay using two genetic mouse models (Rac2^−/−^ [46] and PAD4^−/−^ mice [47]) previously demonstrated to show defective suicidal NETosis. Rac2 is required for oxidative burst-dependent NETosis and the peptidyl-arginine-deiminase 4 (PAD4) is mediating the histone hypercitrullination involved in histone decondensation and subsequent release of NETs. After treatment of neutrophils with PMA or ionomycin, induced NETosis was significantly impaired in Rac2^−/−^ and PAD4^−/−^ mice using both methods of analysis. Furthermore, also human blood-derived neutrophils were used to detect* ex vivo* induced NETs. In summary, this method allows rapid and robust analysis of several thousand cells per sample and is independent of possible biased observer. Since this method is based on the specific staining with antibodies against H3cit, it has the only disadvantage to limit the detection on H3cit-positive events. Since H3cit-deficient mice are not completely deficient in NET formation in response to bacterial pathogens [48], additional H3cit-independent events might be lost using this method.

Zhao et al. described a second flow cytometry technique combined with fluorescence microscopy to quantify NET formation, the so called high-speed multispectral imaging flow cytometry [49]. The authors use transmitted light (brightfield), side-scatter (SSC), and multiple fluorescence images of cellular components (DNA and MPO) to validate nuclear morphology (size, texture, and relative subcellular location). Using this method, vital versus suicidal NETosis can be differentiated. One limitation of this technique is that the system focusses on cells currently undergoing NETosis and may miss cells that have already lysed or are in a late phase of NETosis.

However, both described flow cytometry-based methods allow automated, quantitative, and rapid analysis of neutrophil morphology and introduce the potential for using NETosis as biomarker in preclinical and clinical studies [45, 49].

### 3.5. Electron Microscopy

NETs have also been successfully visualized by transmission electron microscopy (TEM) and scanning electron microscopy (SEM). Both techniques were used by Brinkmann and colleagues for the initial discovery of NETs and TEM by Fuchs et al. to describe the differences between necrosis, apoptosis, and NETosis [3, 34]. While TEM enabled the characterization of the morphology of NET-releasing cells, for example, disintegration of the nuclear membrane [34], SEM demonstrated the structure of NETs. Histones and elastase were detected associated to NETs by SEM-based immunodetection when sampling ultrathin cryosections of IL-8 stimulated neutrophils [3]. Furthermore, SEM demonstrated that NETs are not membrane-bound and have the ability to entrap bacteria [3]. Moreover Krautgartner and Vitkov published a RR-OsO4 staining technique for improved TEM-analysis of the interaction of NETs and bacteria. The authors visualized NETs and fimbriae-mediated bacterial adhesion [50]. In a lot of following studies SEM and TEM were nicely used to characterize the features of NET formation. As an additional selected example, in 2009 mice lacking a functional NADPH oxidase were found to be impaired in NET production compared to respective wildtype mice using EM techniques [51]. However, it has to be mentioned that Remijsen and colleagues questioned the usage of TEM to analyze NETs. The authors observed that fibrin mimics NETs when analyzed with SEM and that a morphological differentiation between NETs and fibrin is untrustworthy in inflammatory exudate samples [52]. Thus, fluorescence microscopy is again needed to verify the results [53].

## 4. *In Vivo* or* In Situ* Staining of NETs and Live-Cell Imaging

Despite the successful visualization of NETs* in vitro*, a lot of scientists in the community still questioned the relevance of NETs during the last years because of the limited available* in vivo* data. Several research groups have successfully stained NETs in fixed tissue sections or blood samples. For this similar immunofluorescence microscopy techniques have been used to show the presence of NETs* in vivo* as shown for* in vitro* visualization of NETs based on DNA-intercalating dyes in combination with antibodies against NET components.

However, one important step for the interpretation of existing data is the definition of the experimental design. In Table 2 we defined our understanding of* in vivo*,* in vitro*,* ex vivo*,* in situ,* and intravital experiments. Furthermore an overview of selected publications focusing on* in vivo* and* in situ* NET detection is given in Table 3.

The first* in situ* staining was conducted in spontaneous human appendicitis where a costaining of neutrophil elastase, histone, and DNA demonstrated NET components inside extracellular fibrous material [3]. In a study about NETs in preeclampsia Gupta and colleagues compared a normal placenta with a preeclamptic placenta and detected an increased NET formation in preeclamptic placenta [54].

As an example for infectious diseases, staining of NETs in mouse skin biopsies was conducted after an infection with an* sda1* deletion mutant of* Streptococcus pyogenes* M1 [23]. In an infection model for* Streptococcus pneumoniae*, NET detection during murine pneumococcal pneumoniae was successful [55]. Besides, the analyses of* Staphylococcus aureus*-infected lungs revealed that the released NETs are decorated with antimicrobial peptides [56].

Most* in vivo*,* in situ*, and* ex vivo* experiments detected less NET fibers compared to results from similar* in vitro* experiments, since the* in vivo* results may be influenced by a complex time-dependent regulatory process of NET induction and NET elimination within the host. However, all available publications contributed to a better understanding of NET function and the role of NETs in the pathogenesis of different diseases. Here some selected publications are summarized.

The method of choice to understand the impact of NETs and ongoing processes during NETosis is the analysis of NET release with live-cell imaging. Already in 2006, Buchanan and colleagues demonstrated a correlation between produced bacterial DNases, NET degradation, and pathogenicity by comparison of histopathological microscopy and live-cell imaging using DNA-intercalating dyes, [23]. Later, in 2007, PMA activated neutrophils were monitored by live-cell imaging over time using a combination of phase contrast, Calcein Blue (vital dye), Annexin V (death marker), and histone marker. This live imaging study led to the conclusion that neutrophils die an active cell death when releasing NETs [34]. Additionally, the release of mitochondrial DNA from viable neutrophils was monitored by live-cell imaging using a mixture of different DNA-intercalating dyes [39]. Several other examples investigated the NET release after neutrophil stimulation by live-cell fluorescence microscopy focussing on host-pathogen interaction [57], biochemical processes, and pathway steps during NETosis [53, 58–61].

A special case for an* in situ* NET detection is the live-cell imaging performed immediately after euthanasia of the animals without a fixation of selected organs. Using two-photon microscopy in* Aspergillus fumigatus* infected lungs, NETs were nicely detected in real time* in situ* [57]. Therefore the lung lobes of infected mice were prepared, dissected, and directly visualized with SYTOX dye. During the live imaging, the lung was embedded in PBS at 37°C. Another interesting example is the analysis of body fluids promptly after the withdrawal from patient material which allows* ex vivo* analysis of NETs. Using this approach, NETs were detected in synovia from patients with gouty [14].

Finally, the real-time documentation of a viable NET-forming cell* in vivo* by intravital microscopy was a giant step in the investigation of NET formation. In 2007, Clark et al. demonstrated that neutrophils release NETs in response to platelet activation using intravital microscopy of the liver sinusoid [38]. Further intravital studies demonstrated the NET formation in the carotid bifurcation using two-photon microscopy [62, 63], in the liver sinusoids using spinning disk confocal microscopy [64], and intravenously using two-photon with epifluorescence microscopy [65]. As mentioned above, based on intravital techniques Yipp and colleagues hypothesized that viable neutrophils release NETs by a vesicular process. To verify this hypothesis they combined an* in vivo* experiment with spinning disk confocal intravital microscopy [35]: exteriorized mouse skin was infected with* Staphylococcus aureus* and monitored for NET formation over 2 hours. The NET detection was conducted with SYTOX dye. From this study it was concluded that NET-forming neutrophils are not immediately dead and the authors claimed the existence of the abovementioned two different mechanisms of NET formation, the suicidal versus vital NETosis [36].

Lately, an intravascular detection of NETs in the bloodstream was successfully confirmed in septic mice [64] using a similar protocol as previously described by Clark and colleagues [38]. Immunofluorescence labeled* Escherichia coli* were detected inside NETs using spinning disk confocal intravital microscopy (SD-IVM). In addition Tanaka and colleagues characterized NETs in blood vessels of different organs using intravital microscopy with a laser-scanning microscope [66]. Thus, the combination of different live-cell imaging methods with* in vivo* or* in situ* experiments has been widely used as an excellent method to monitor the NET release in case of an infection or autoimmune disease.

## 5. Conclusions and Perspectives

Actual knowledge indicates that NET formation is a complex phenomenon mediated by a highly specialized population of neutrophils in response to infection and inflammation. During the last years, especially intravital microscopy techniques facilitated the* in vivo* evaluation of the role of NETs during several diseases. However, to fully understand the mechanisms mediating NET formation and the role of NETs during health and disease, improved NET visualization and quantification techniques are needed.

Since the simple visualization and quantification of NETs using DNA-intercalating dyes has the risk of detection of necrotic cells or the generation of artificial results based on dye-blocking peptides associated with NETs, antibody-based techniques are definitely needed to visualize NETs. In general, microscopy is very specific but always has the limitation to be observer dependent and very time-consuming. Thus, there is a need for methodologies that enable robust and rapid assessment of NET-releasing cells, for example, by flow cytometry combined with imaging. Flow cytometry-based techniques include the advantage to enable further sorting of selected cell populations, for example, to differentiate between cells that undergo vital versus suicidal NETosis. In our opinion, future experiments should especially focus on single cell analysis to characterize the detailed cellular events that mediate formation of NETs in individual cells of a neutrophil population [41]. This may help to differentiate the specific signaling processes that lead to the different phenotypes of NET formation compared to other antimicrobial strategies as phagocytosis or degranulation. For this, special techniques, for example, flow cytometry-based sorting or laser dissection methods, are needed.

## Figures and Tables

**Table 1:** Summary of the main NET visualization techniques used for quantification of NETs and its advantages or disadvantages.

Dye	Technique	Parameter	Advantages	Disadvantages	Selected references
SYTOX dye/PicoGreen	FM, eye	Percentage of NET formation	Visible differentiation between necrosis and NETosis	Occasionally biased by selection of field of view, staining of DNA in NETs by DNA-intercalating dye can be blocked by cationic peptides	[3, 40, 44]

Antibody against histone-DNA complexes + Dapi	IFM, eye	Percentage of NET formation	Visible differentiation between necrosis and NETosis	Occasionally biased by selection of field of view	[7, 8, 10, 34, 40, 44]

Antibody against elastase and histone-DNA complexes + Hoechst 33342	IFM, Image J	Percentage of NET formation	Unbiased software-based quantification	Clump of NETs derived from multiple cells count as one single event, occasionally biased by selection of field of view	[67]

Antibody against histone-DNA complexes + Dapi	IFM, Image J	Level of NET degradation	Unbiased software-based quantification	Occasionally biased by selection of field of view	[24, 25]

Antibody against histone-DNA complexes + Dapi	IFM, open source software	Level of NET degradation	Unbiased software-based quantification	Occasionally biased by selection of field of view	[68]

SYTOX dye/PicoGreen	FR	DNA release (*μ*g/mL)	Unbiased	No differentiation between necrosis and NETosis, staining of DNA in NETs by DNA-intercalating dye can be blocked by cationic peptides	[27, 37, 40]

PicoGreen after nuclease digestion	FR	DNA release (*μ*g/mL)	Unbiased	Staining of DNA in NETs by DNA-intercalating dye can be blocked by cationic peptides, less sensitive compared to antibody-mediated detection of NETs	[34, 40]

Antibody against MPO + Hoechst	Imaging flow cytometry	Percentage of NET formation	Unbiased, automated, enables differentiation between suicidal NETosis and vital NETosis	Imaging of cells currently undergoing NETosis and thus this method may miss those that have already lysed	[49]

Antibody against H3cit + MPO	Flow cytometry	Percentage of NET formation	Unbiased, automated, can be combined with sorting	Does not detect H3cit-independent events	[45]

Uranyl-acetate, osmium tetroxide, ruthenium red-osmium tetroxide, Cuprolinic Blue	TEM	Morphology of NET-releasing cells	Visible differentiation between necrosis and NETosis, can be used in combination with immunostaining of certain structures in NETs	Occasionally biased by selection of field of view	[40, 50]

Osmium tetroxide/gold	SEM	Amount and structure of NETs-releasing cells	Visible differentiation between necrosis and NETosis, can be used in combination with immunostaining of certain structures in NETs	Occasionally biased by selection of field of view, fibrin mimics NET structures	[40, 50]

IFM: immunofluorescence microscopy, FM: fluorescence microscopy, FR: fluorescence reader, MPO: myeloperoxidase, TEM: transmission electron microscopy, SEM: scanning electron microscopy, and H3cit: histone citrullination.

**Table 2:** Definition of Latin terms used in this paper for the detection of NETs in viable or fixed samples and individuals.

Latin term	Translation	Definition used in this review	Examples
*In vivo*	Within the living organism	Effects analyzed on whole, living organisms	Detection of NETs in a living animal after an infection with bacteria

*In situ*	In the natural, correct position	Effects analyzed on whole or partial organisms that are dead	Detection of NETs in histological samples derived from infected animals

*Ex vivo*	Out of the living organism	Effects analyzed on live isolated cells or biopsies with a minimum of changes of the natural conditions (tissue or body fluid)	Detection of NETs in body fluids (cytospin) or in tissue without fixation directly after isolation from viable organism

*In vitro*	In a test tube	Effects tested under laboratory conditions in test tubes, for example, Petri dishes	Detection of NET formation in response to pathogens or chemicals by purified blood-derived neutrophils in tissue culture plates

Intravital	Occurring during life	Visualization of an event in the living organisms by microscopy, same as *in vivo*	See *in vivo*

**Table 3:** Overview of selected examples for *in vivo* and *in situ* detections of NETs.

References	*In vivo*	*In situ*	Species	Pathogen/disease	Organ/tissue/body fluid	Method(s)
[3]		x	Rabbit	Experimental shigellosis	Intestine	Immunofluorescence microscopy
[3]		x	Human	Spontaneous appendicitis	Intestine	Immunofluorescence microscopy
[54]		x	Human	Preeclampsia	Placentae	Immunofluorescence microscopy
[55]		x	Murine	*Streptococcus pneumoniae*/pneumonia	Lung	Immunofluorescence confocal microscopy
[23]		x	Murine	Group A *Streptococcus*/necrotizing fasciitis	Skin	Immunofluorescence microscopy
[38]	x		Murine	Endotoxemia model (LPS induced)	Liver	Intravital microscopy
[69]		x	Human	*Plasmodium falciparum*	Peripheral blood	Cytology
[70]		x	Human	Small-vessel vasculitis (SVV)	Kidney	Immunofluorescence microscopy
[17]		x	Murine	*Candida albicans*	Skin, lung	Immunofluorescence microscopy
[17]		x	Murine	*Candida albicans*	Lung	Scanning electron microscopy
[71]		x	Human	Periodontitis	Crevicular exudate samples, pocket epithelium biopsies	Immunofluorescence microscopy, histology microscopy, scanning electron microscopy, transmission electron microscopy
[72]		x	Human	*Leishmania amazonensis/*Cutaneous Leishmaniasis	Skin	Immunofluorescence microscopy
[15]		x	Baboon	Thrombosis (deep vein thrombosis)	Vein	Immunohistochemistry
[56]		x	Murine	*Staphylococcus aureus*	Lung	Immunofluorescence confocal microscopy
[57]		x	Murine	*Aspergillus fumigatus/*pneumonia	Lung	Two-photon microscopy
[73]		x	Human	Systemic lupus erythematosus	Skin, kidney	Immunofluorescence microscopy
[8]		x	Murine	Influenza/pneumonia	Lung	Immunofluorescence microscopy, histology microscopy
[62, 63]	x		Murine	Atherosclerosis	Carotid artery	Intravital two-photon microscopy
[62]		x	Human	Atherosclerosis	Carotid artery	Immunohistochemistry
[64]	x		Murine	Sepsis (LPS, *Escherichia coli, Staphylococcus aureus*)	Liver	Spinning disk confocal intravital microscopy
[74]		x	Murine	Lung injury (LPS)	Lung	Immunofluorescence microscopy
[35]	x		Murine/human neutrophils in viable mouse	*Staphylococcus aureus* induced abscess	Skin	Spinning disk confocal intravital microscopy
[35]		x	Human	*Staphylococcus aureus* induced abscess	Skin	Transmission electron microscopy
[65]	x		Murine	Thrombosis	Vein	Intravital two-photon microscopy
[65]		x	Murine	Thrombosis	Vein	Immunohistochemical microscopy
[75]	x		Murine	Tumor	Liver, lung	Spinning disk confocal intravital microscopy
[75]		x	Murine	Tumor	Liver, lung	Immunofluorescence confocal microscopy
[76]		x	Murine	*Streptococcus pneumoniae* and influenza/pneumonia	Lung	Immunofluorescence microscopy
[66]	x		Murine	Sepsis model (LPS)	Blood, liver, lung	Intravital imaging for intra-abdominal organs using a multiphoton microscope
[14]		x	Human	Gouty	Joint, cytospin synovial fluid	Immunofluorescence microscopy
[14]		x	Murine	MSU induced aggNETs	Intraperitoneal aggregates	Immunohistochemical microscopy
[77]	x		Murine	*Staphylococcus aureus*	Liver	Spinning disk confocal intravital microscopy

## Abbreviations

NET: Neutrophil extracellular trap
PMA: Phorbol-12-myristate-13-acetate.
